# The Role of Sex Differences in the Link Between Emotion Regulation and Psychological Well-Being During a Major Mental Health Crisis

**DOI:** 10.3390/bs15050636

**Published:** 2025-05-07

**Authors:** Zeyi Zang, Florin Dolcos, Kelly Hohl, Paul C. Bogdan, Sanda Dolcos

**Affiliations:** 1 Department of Psychology, University of Illinois Urbana-Champaign, Champaign, IL 61820, USA; hohl2@illinois.edu (K.H.); paul.bogdan@duke.edu (P.C.B.); 2 Beckman Institute for Advanced Science & Technology, University of Illinois Urbana-Champaign, Urbana, IL 61801, USA; 3 Neuroscience Program, University of Illinois Urbana-Champaign, Urbana, IL 61801, USA

**Keywords:** gender, cognitive reappraisal, expressive suppression, distress, resilience

## Abstract

Emotion regulation (ER) strategies, such as reappraisal and suppression, have been linked to psychological well-being. The available evidence points to the differential impact of ER strategies on resilience and post-traumatic growth (PTG), as factors related to well-being, as well as to sex differences in the link between ER preference and well-being. However, previous studies are mixed regarding these links. To address this issue, college students (N = 1254) recruited between 2020 and 2023 reported their habitual use of ER strategies, resilience and PTG during the COVID-19 pandemic, which, as a global health crisis, has raised not only severe physical health concerns but also mental distress. First, reappraisal was positively associated with both resilience and PTG, whereas suppression was negatively correlated with these measures. Second, female participants had lower suppression scores and higher PTG scores than male participants. Third, a moderation analysis showed that the positive relationship between reappraisal and PTG was stronger in female participants, whereas the negative relationship between suppression and PTG was stronger in male participants. Overall, these findings shed light on the links among ER strategies, resilience, and PTG and have relevance for customized training in the use of reappraisal to increase well-being in women and men.

## 1. Introduction

Unlike the exposure to acute traumatic events, the experience of continuous traumatic distress, such as that associated with chronic illness or major mental health crises, is a unique and atypical type of trauma that can lead to severe consequences (Pat-Horenczyk et al., 2013; Whittemore & Dixon, 2008). Nevertheless, evidence about how people deal with and recover from such chronic stressful events is limited. The COVID-19 pandemic, which has raised significant global psychological health concerns, has provided a historically valuable context for researchers to study the factors that protect people from such impactful chronic mental challenges. Among those who were negatively impacted by the pandemic, college students reported significantly higher mental distress, including anxiety and depression, than the general population (Romeo et al., 2021). On the one hand, given the vulnerability of college students, their increased levels of perceived stress and concerns about the future throughout the pandemic were shown to be risk factors that negatively impacted their psychological well-being (Quintiliani et al., 2021; Arsandaux et al., 2020). On the other hand, emotion regulation (ER) strategies, such as reappraisal, have been proposed as ways of improving psychological well-being. Although the available evidence points to differential impact of ER strategies on resilience and post-traumatic growth (PTG), which are factors related to well-being that reflect recovery and growth, respectively, after distress, previous studies are mixed regarding these links. Therefore, the overarching goal of the current study was to identify which ER strategies used by college students during the pandemic had a protective effect and contributed to enhanced resilience and PTG. Given the evidence of sex differences in the link between the preferred use of certain ER strategies and well-being (Rogier et al., 2017), the present study also compared the use and impact of ER strategies in women and men.

Emotion control strategies are processes that modulate one’s emotion to adapt to a given situation and have been closely related to resilience and PTG (Eze et al., 2022; Dolcos et al., 2021). The focus here was on two ER strategies that are typically employed in everyday life: cognitive reappraisal and expressive suppression. Reappraisal involves the reinterpretation of negative stimuli or situations in a more positive or adaptive way and has shown beneficial effects (Gross, 1998). Reappraisal plays a significant protective role against anxiety and depression (Llewellyn et al., 2013; Dolcos et al., 2021) and has been linked to higher levels of resilience (Han et al., 2023) and PTG (Kim et al., 2023; Orejuela-Dávila et al., 2019). These beneficial effects emphasize the important role of reappraisal in supporting positive changes during and after traumatic experiences. Expressive suppression involves inhibiting the expression of emotions, typically following exposure to negative stimuli or situations, and its habitual engagement is positively associated with symptoms of distress (Gross, 1998; Llewellyn et al., 2013). However, the evidence regarding the link between suppression and the aspects of well-being targeted in the present study—resilience and PTG—is mixed, with some studies showing a beneficial effect and others detrimental or no effects. Consistent with a beneficial effect, the suppression of negative emotion has been shown to be positively correlated with PTG (Jiang et al., 2022) and with decreased anhedonia symptoms in depressed youth (Young et al., 2022; reviewed in Troy et al., 2023). Consistent with a maladaptive effect, there is evidence of negative associations between suppression and PTG (Eze et al., 2022) and between suppression and resilience (Mouatsou & Koutra, 2023). Nonetheless, other studies have not found any link between the use of suppression and resilience (e.g., Kim et al., 2023). Therefore, more evidence is needed to clarify the association between ER and psychological well-being and to determine whether suppression is an adaptive or maladaptive ER strategy during chronic exposure to distress. In turn, this will inform exploration of effective strategies to enhance one’s well-being during chronic emotional challenges.

To understand the effect of reappraisal and suppression on psychological well-being, it is also important to specifically define and distinguish between the two aspects of well-being targeted by the present study: resilience and PTG. Resilience is defined as the ability to maintain a relatively stable mental state and bounce back quickly after experiencing aversive events (Bonanno, 2004), and PTG reflects positive adaptive changes after distressing events (Tedeschi & Calhoun, 2004). Although resilience and PTG were found to be closely linked (Duan et al., 2015; Widyorini et al., 2022), with some researchers arguing that resilience contains key features of PTG, such as growing from distress (den Hartigh & Hill, 2022), there is also evidence that these two factors are distinct. Resilience reflects the ability to retain healthy functioning after aversive experiences (Bonanno, 2004), whereas PTG more strongly emphasizes the transformations following struggles with stressors and is concerned less about the level of individual adaptiveness before traumatic experiences (Tedeschi & Calhoun, 2004). By evaluating these two distinct, yet related, aspects of psychological well-being, the present study aimed to more comprehensively examine the role of ER in facing the challenges posed by the pandemic.

Resilience and PTG have proven helpful in adapting to adverse situations, including experiences associated with major crises (Ala et al., 2024; Rapport et al., 2020; Kiltz et al., 2023; Li & Hu, 2022). During the COVID-19 lockdown period, resilience and PTG played an important role in helping people to effectively recover and grow positively from this stressful period. For example, there is evidence that individual resilience reduced the stressful experience in the pandemic period (Kimhi et al., 2020). Additionally, PTG was found to be negatively correlated with pandemic-related stress (Di Corrado et al., 2022) and positively predicted well-being during the pandemic (Kiltz et al., 2023). Considering the positive effect of resilience and PTG in the lockdown period, it is reasonable to expect that they have had a protective role against stressors throughout the COVID-19 pandemic. Hence, one main goal of the present study was to clarify the link between resilience and PTG, as aspects of well-being, and the habitual use of reappraisal and suppression, as ER strategies, among college students, during the COVID-19 pandemic.

There is also evidence of sex differences in ER choice, resilience and PTG, which may impact the associations among these measures in college students. For example, there is evidence that women tend to habitually use reappraisal more than men, who tend to use suppression more than women (Gross & John, 2003; Flynn et al., 2010; Rogier et al., 2017; reviewed in Nolen-Hoeksema, 2012). These sex differences in the habitual use of ER strategies, together with the positive associations between reappraisal and resilience and PTG, along with the potential negative impact of suppression on them, may suggest a higher level of resilience and PTG in women than in men. Nevertheless, the available evidence points to a more complex effect of ER on resilience and PTG. Specifically, there is evidence that men show higher levels of resilience than women after experiencing traumatic events, including the distress associated with the COVID-19 pandemic (Stratta et al., 2013; Yan et al., 2021; Peyer et al., 2022). On the other hand, although women are more vulnerable to risk factors that lead to traumatic experiences than men (García-Fernández et al., 2020), they also tend to show a higher level of PTG (Jin et al., 2014), including during the recent pandemic (Cohen-Louck, 2022). Therefore, further clarification is needed regarding the role of sex differences in the effect of ER on resilience and PTG, which was the second main aim of the present study. Based on this, more individualized ER strategies can be deployed, to reduce distress more effectively in women and men.

To summarize, the present study aimed to clarify the links between the habitual use of two ER strategies (reappraisal and suppression) and two aspects of psychological well-being (resilience and post-traumatic growth), and to investigate the role of sex differences in these associations. The following three hypotheses were tested:

(1) Reappraisal was positively associated with resilience and PTG, whereas suppression was negatively linked to resilience and PTG, during the pandemic.
(2) Female participants used reappraisal more, whereas male participants used more suppression more, as ER strategies during the COVID-19 pandemic. Possible sex differences in resilience and PTG scores were also investigated.
(3) Given the evidence that the choice of ER differs in men and women, it is possible that the links between ER and aspects of well-being are differential across sex groups. Hence, the possibility that sex could moderate the link between ER strategies and resilience and PTG was also explored. Namely, it was tested whether the positive association between reappraisal and resilience or PTG was stronger in female participants, and whether the negative association between suppression and resilience or PTG was stronger in male participants.

## 2. Methods

### 2.1. Participants

A total of 1564 college students aged between 18 and 31 (69.8% females, 28.4% males, 1.8% others; 37.9% White, 33.6% Asian, 17% Hispanic, 10.6% Black) were recruited through the University of Illinois at Urbana-Champaign (UIUC) Psychology subject pool (SONA), between 2020 and 2023 (see Procedures and Questionnaires Section below). The demographic information was collected based on self-reported details provided by participants in response to prompts regarding their sex/gender (1 = Male; 2 = Female; 3 = Transgender; 4 = Non-binary; 5 = Other; 6 = Prefer not to answer) and racial identity (1 = White, not of Hispanic origin; 2 = First Nations origin; 3 = Asian or Pacific Islander; 4 = Hispanic; 5 = Black, not of Hispanic origin). Datasets from 310 participants were excluded from the analyses due to incomplete or low-quality answers (N = 241), failed comprehension checks (N = 47), or limited sample size in some gender groups (N = 22). Thus, the present findings are based on data from 1254 participants (71% females, 29% males; 37.6% White, 33.9% Asian, 17.1% Hispanic, 10.7% Black). The number of datasets slightly varied across different measures and was reflected in the degrees of freedom reported analysis-wise. The final sample contained 943 datasets for the emotion regulation scale, 1192 for the resilience scale and 1254 for the PTG scale. Given that in all cases the Ns are high, these differences are not concerning. Sensitivity analyses demonstrate that this sample has sufficient power (80%) to detect significant (α = 0.05) differences between means (independent t-tests of equal sized groups) of *d* > 0.15 and correlations of *r* > 0.08. Therefore, the sample size is well powered for detecting even small effects. The surveys and procedures used in the current study were approved by Institutional Review Board (IRB) at UIUC, and all participants were compensated for their time with course credits.

### 2.2. Procedures and Questionnaires

There were no inclusion or exclusion criteria for participating in this study because all students with access to the study through the UIUC SONA system were within the population of interest. All participants provided online informed consent prior to data collection, completed online surveys administered through Qualtrics, and then were compensated for their time with course credits. To address the present questions, participants completed the following three questionnaires: Emotion Regulation Questionnaire (ERQ), Connor-Davidson Resilience Scale (CD-RISC) and Post-Traumatic Growth Inventory (PTGI).

### 2.3. Emotion Regulation

The Emotion Regulation Questionnaire (Gross & John, 2003) was used to assess the habitual use of two emotion regulation strategies: cognitive reappraisal (6 items; e.g., “*I control my emotions by changing the way I think about the situation I am in*”; α = 0.86) and expressive suppression (4 items; e.g., “*I control my emotions by not expressing them*”; α = 0.77). Participants rated each item on a 7-point scale (1 = “*Strongly disagree*”; 7 = “*Strongly agree*”). The ratings of each subscale were summed up to obtain the scores for reappraisal and suppression, respectively, which were used separately in the analyses.

### 2.4. Resilience

The Connor–Davidson Resilience Scale (Connor & Davidson, 2003) was used to evaluate resilience, conceptualized as a measure of stress coping abilities and qualities that enable one to thrive in the face of adversity (Connor & Davidson, 2003). The scale consists of 25 items assessing how quickly participants bounce back after experiencing a negative event (e.g., “*Able to adapt to change*”; α = 0.94), using a 5-point scale (1 = “*Not true at all*”; 5 = “*True nearly all of the time*”). An overall score of resilience level for each participant was obtained by summing up the rating of each item (higher scores indicate higher levels of resilience).

### 2.5. Post-Traumatic Growth

The Post-Traumatic Growth Inventory (Tedeschi & Calhoun, 1996) was used to assess the level of PTG. The PTGI scale consists of 21 items that assess changes in peoples’ lives following a traumatic experience, crisis or a highly challenging situation (α = 0.87). Each item consisted of a statement describing a potentially positive change in one’s mental state linked to the crisis (e.g., “*I am better able to accept the way things work out*”). Participants rated the extent to which they believed that the statement applied to them using a 6-point scale (1 = “*I did not experience this change*”; 6 = “*I experienced this change to a very great degree*”). An overall score of PTG level was obtained by summing up the rating of each item (higher scores indicate higher levels of PTG).

### 2.6. Data Analyses

**Figure 1:**
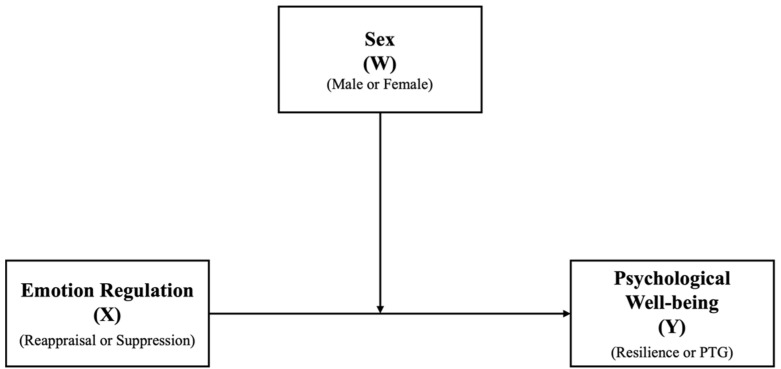
Conceptual moderation model. The interaction effect of sex (W) on the association between the use of emotion regulation (X) and the level of psychological well-being (Y) was examined.

The associations between ER (e.g., reappraisal and suppression) and psychological well-being (e.g., resilience and PTG) were tested using Pearson’s correlation (significance level of *p* < 0.05, two-tailed). The sex differences in the scores of the four variables of interest—reappraisal (ERQ-R), suppression (ERQ-S), resilience (CD-RISC) and PTG (PTGI)—were examined using independent-sample t-tests (significance level of *p* < 0.05, two-tailed). Finally, the role of sex differences in the links between reappraisal and suppression and resilience and PTG were examined by moderation models using the Hayes PROCESS macro (Model 1). The interactions between the independent (e.g., reappraisal and suppression) and the moderator (e.g., male vs. female) variables led to four individual moderated regression models (see conceptual model in Figure 1). The reliability of measures was assessed using Cronbach’s Alpha, and the results indicated that all measures had good internal consistency (α > 0.7). All statistical analyses were conducted using SPSS for MacBook (IBM, Version 29.0).

## 3. Results

### 3.1. Divergent Associations of Reappraisal and Suppression with Resilience and Post-Traumatic Growth

**Figure 2:**
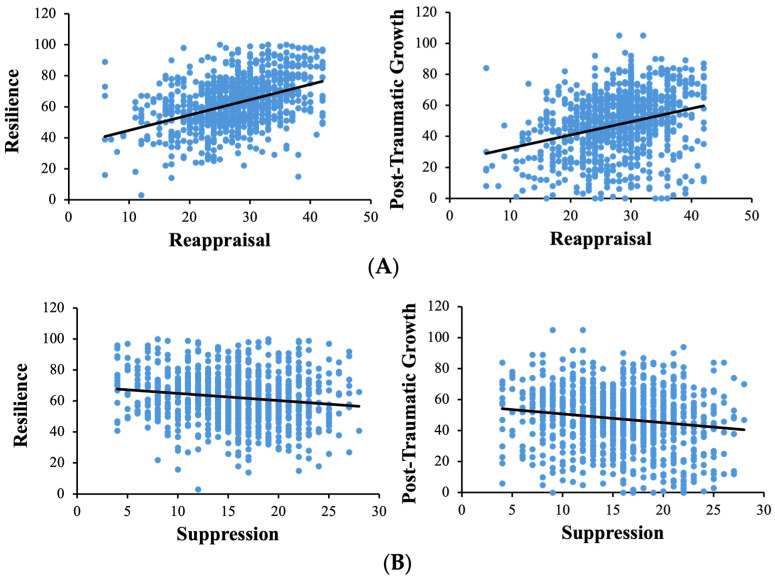
Divergent associations of reappraisal and suppression with resilience and post-traumatic growth. (**A**) Positive correlations of reappraisal with resilience and PTG scores; (**B**) negative correlations of suppression with resilience and PTG scores.

Confirming our first hypothesis, there were opposing associations of reappraisal and suppression with resilience and PTG (Table 1 and Figure 2). As illustrated in Figure 2A, the habitual use of reappraisal (ERQ-R) was positively associated with resilience (CD-RISC; *r* = 0.427, *p* < 0.001) and PTG (PTGI; *r* = 0.302, *p* < 0.001). In contrast, as shown in Figure 2B, the habitual use of suppression (ERQ-S) was negatively associated with resilience (CD-RISC; *r* = −0.150, *p* < 0.001) and PTG (PTGI; *r* = −0.151, *p* < 0.001).

### 3.2. Sex Differences in Suppression and PTG

**Figure 3:**
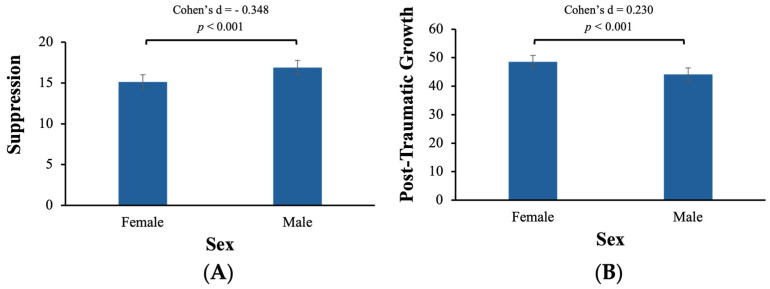
Sex differences in suppression (**A**) and post-traumatic growth (**B**).

Partially confirming the second hypothesis, our findings showed sex differences in the levels of suppression and PTG. As illustrated in Figure 3A, male participants (*M* = 16.9 *SD* = 5.19) tended to use more suppression than female participants (*M* = 15.1 *SD* = 4.63) (*t*[941] = 4.86, *p* < 0.001). However, different from the hypothesis, there were no significant sex differences in the habitual use of reappraisal (*t*[941] = 0.664, *p* = 0.507), nor in the reported level of resilience (*t*[1190] = 0.502, *p* = 0.616). Additionally, as shown in Figure 3B, male participants (*M* = 44.2 *SD* = 19.3) also reported a lower level of PTG than female participants (*M* = 48.6 *SD* = 19.4; *t*[1252] = −3.70, *p* < 0.001).

### 3.3. Sex Moderated the Link Between ER and PTG

**Figure 4:**
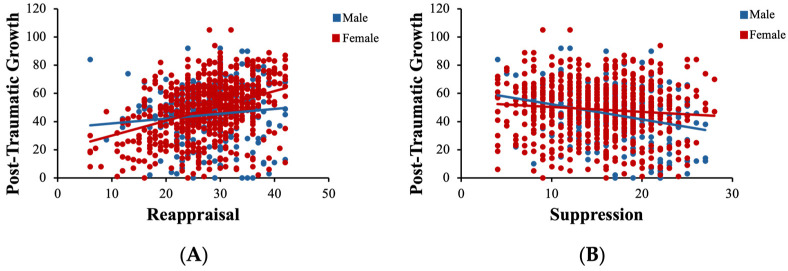
Sex moderated the link between reappraisal and PTG (**A**) and between suppression and PTG (**B**). (**A**) Individual regression analyses within respective sex clusters indicated significant slope for females (*p* < 0.001), but not for males (*p* = 0.055). (**B**) Individual regression analyses within respective sex clusters indicated significant slopes for both females (*p* = 0.014) and males (*p* < 0.001).

Partially confirming the third hypothesis, sex moderated the associations of reappraisal and suppression with post-traumatic growth. First, as Figure 4A illustrates, the positive link between reappraisal and PTG (Figure 2A) was stronger in female than in male participants. By adding the interaction term (reappraisal × sex), the model significantly predicted PTG [Δ*R*^2^ = 0.013, *F*(1, 939) = 9.46, *p* = 0.002]. Individually, reappraisal positively predicted the level of PTG (*β* = 0.118, *SE* = 0.058, *p* = 0.041, η^2^ = 0.09), and sex also served as a positive predictor (*β* = 0.201, *SE* = 0.066, *p* = 0.002, η^2^ = 0.01). Second, as shown in Figure 4B, the negative relationship between suppression and PTG (Figure 2B) was stronger in male than in female participants. By adding the interaction term (suppression × sex), the model significantly predicted PTG [Δ*R*^2^ = 0.007, *F*(1, 939) = 6.67, *p* = 0.016]. Individually, suppression negatively predicted the level of PTG, *β* = −0.281, *SE* = 0.064, *p* < 0.001, η^2^ = 0.02, and sex did not serve as a significant predictor, *β* = 0.114, *SE* = 0.071, *p* = 0.108. Nonetheless, sex did not play a moderating role in the relation between reappraisal and resilience (*F*(1, 939) = 2.024, *p* = 0.153), nor between suppression and resilience (*F*(1, 939) = 0.0004, *p* = 0.986).

## 4. Discussion

### 4.1. Divergent Associations of Reappraisal and Suppression with Resilience and Post-Traumatic Growth

The positive association between reappraisal and both resilience and PTG is consistent with evidence showing an adaptive effect of habitually using reappraisal (e.g., Dolcos et al., 2021; Orejuela-Dávila et al., 2019) and expands the evidence regarding its beneficial effects when facing major emotional distress. As a strategy focused on engaging with and changing the meaning of the emotional content, reappraisal might promote constructive thinking and thus enable individuals to engage with trauma-related emotions and memories (Tedeschi & Calhoun, 2004). This is consistent with recent research suggesting that strategies that involve engagement with emotional stimuli influence PTG by helping individuals to extract meaning from their traumatic experiences (Larsen & Berenbaum, 2015). The evidence from the current study supports former findings linking reappraisal to college students’ resilience and PTG under the COVID-19 pandemic (Mouatsou & Koutra, 2023; Li & Hu, 2022).

The negative associations of suppression with resilience and PTG in the present study are consistent with evidence regarding the maladaptive effects of habitual engagement of this ER strategy on symptoms of distress (Llewellyn et al., 2013) and on resilience and PTG (e.g., Mouatsou & Koutra, 2023; Eze et al., 2022). Expressive suppression, a response-focused strategy that involves changing the behavioral expression of emotions, is thought to be less effective long term than antecedent-focused strategies, such as reappraisal, because it cannot prevent the generation of fully developed negative affective responses (Gross & John, 2003). While the role played by suppression in the modulation of mental distress is mixed in the literature, suppression has been found to closely connect with a wide range of stress-related symptoms, such as anxiety and post-traumatic stress, among individuals exposed to traumatic events (Moore et al., 2008; reviewed in Dryman & Heimberg, 2018). Hence, the present findings add to the literature regarding the disadvantage of habitually using this ER strategy and extend the evidence regarding its maladaptive effects on resilience and PTG when facing major emotional challenges, such as the COVID-19 pandemic.

### 4.2. Sex Differences in Suppression and Post-Traumatic Growth

The present finding that male participants were more likely to use suppression than female participants is also consistent with previous studies (e.g., Rogier et al., 2017; Jin et al., 2014). The fact that sex differences in the use of suppression persisted during the pandemic is notable and suggests that men’s tendency to use suppression more than women do is stable and persists even in the face of universally experienced stressors. The increased levels of PTG in women are also in line with previous studies (e.g., Jin et al., 2014; Cohen-Louck, 2022), which have attributed this difference to a variety of factors, including women’s tendency to use more adaptive ER strategies, such as reappraisal. However, different from the previous evidence, our study does not show sex differences in the levels of reappraisal. It is possible that during the pandemic, which exposed the whole population to a prolonged and uncontrollable stressor, women found it harder to look at stressful experiences in a positive way. A more effective use of reappraisal, along with the potential use of other adaptive strategies, such as emotional and social support, may have contributed to higher levels of PTG in female compared to male participants in the present study.

The absence of differences in the resilience levels between women and men in the present study is inconsistent with previous evidence (e.g., Stratta et al., 2013; Peyer et al., 2022), although, in general, female participants tended to report higher level of anxiety and stress during the pandemic (Bigalke et al., 2020; Yan et al., 2021). Overall, while our findings provide additional supporting evidence of sex differences in the level of suppression and PTG, there were no significant differences between women and men in the level of reappraisal and resilience.

### 4.3. Sex Moderated the Link Between Emotion Regulation and Post-Traumatic Growth

The moderation models investigating sex differences showed that, whereas all participants benefited from using reappraisal, in general, women benefited more from using reappraisal, which was more strongly associated with higher PTG scores than in men. By contrast, although resilience and PTG levels were negatively associated with suppression in both male and female participants, the negative association between suppression and PTG was stronger in men than in women. Combined with the present finding of a higher preference for using suppression in male participants, the negative association between this ER strategy and PTG points to a stronger maladaptive effect of using suppression on aspects of well-being, in men, when facing major chronic distress.

Finally, neither the link of reappraisal nor that of suppression with resilience differed across the sex groups, suggesting their generalized opposing associations (positive vs. negative, respectively) in women and men, during the pandemic. These finding are not consistent with previous claims that women tend to use ER less effectively or show lower levels of resilience than men after stressful experiences (McRae et al., 2008). Moreover, although there is evidence regarding the protective role of reappraisal against psychological distress or the detrimental effect of suppression on psychological well-being during the COVID-19 pandemic (e.g., Ursu & Măirean, 2022; Ye et al., 2022), our evidence further shows that the link between these two ER strategies and resilience was similar in women and men during the pandemic. Overall, the present results provide novel insight into the similarities and differences in women and men regarding the link between the ER choices and aspects of well-being during chronic distress. These findings have relevance for the possible customization of training in the use of reappraisal in women and men, to increase its effectiveness when facing emotional challenges.

#### Caveats

Notwithstanding the relevance of our findings, the following three limitations should also be noted. First, because of its cross-sectional nature, our study did not capture changes in the individuals’ preference for using ER strategies and their associations with resilience and PTG across time. Hence, the present findings should be confirmed in future studies involving longitudinal design, by collecting data from the same participants at various stages of a chronic distressing event. Second, our sample involved only college student participants, which reduces the generalizability of our findings. Thus, it is essential to extend the present findings to a more diverse sample. Finally, although the present study measured two typical ER strategies, a wider range of ER and coping strategies would provide a more comprehensive view about the link between ER and well-being.

## 5. Conclusions

The current study shed light on the links between emotion control and psychological well-being, as well as demonstrating the important role of sex differences in the use of ER strategies during a major global challenge. First, reappraisal was positively associated with both resilience and PTG, whereas suppression was negatively associated with these two aspects of well-being. Second, female participants had lower suppression scores and higher PTG scores than male participants. Third, a moderation analysis showed that the positive relationship between reappraisal and PTG was stronger in women, whereas the negative relationship between suppression and PTG was stronger in men. Specifically, while the habitual use of reappraisal was associated with a higher PTG level in both male and female participants, male participants showed a lower PTG level when using more reappraisal, compared to female participants. In contrast, whereas suppression was associated with a lower PTG level in both male and female participants, male participants tended to show a lower PTG level when using more suppression, compared to female participants. These findings have relevance for customized training in the use of reappraisal in women and men, to increase well-being. The identified links deserve consideration for the identification of optimal ER strategies both when facing everyday emotional challenges and when experiencing distress associated with major crises.

## Figures and Tables

**Table 1:** Correlations among the main measures involved.

Variables	Mean	SD	Range	1	2	3	4
1. Reappraisal	27.63	6.757	[6, 42]	--			
2. Suppression	15.65	5.092	[4, 28]	0.030	--		
3. Resilience	62.75	15.618	[3, 100]	0.427 ***	−0.150 ***	--	
4. PTG	47.69	19.041	[0, 105]	0.302 ***	−0.151 ***	0.470 ***	--

*** Significant at *p* < 0.001.

## Data Availability

The data will be made available to interested scientists, upon requests addressed to the authors, following article acceptance and IRB approval for data sharing.
